# Single Nucleotide Polymorphisms of Human STING Can Affect Innate Immune Response to Cyclic Dinucleotides

**DOI:** 10.1371/journal.pone.0077846

**Published:** 2013-10-21

**Authors:** Guanghui Yi, Volker P. Brendel, Chang Shu, Pingwei Li, Satheesh Palanathan, C. Cheng Kao

**Affiliations:** 1 Department of Molecular and Cellular Biochemistry, Indiana University, Bloomington, Indiana, United States of America; 2 Department of Biology, Indiana University, Bloomington, Indiana, United States of America; 3 School of Informatics and Computing, Indiana University, Bloomington, Indiana, United States of America; 4 Department of Biochemistry & Biophysics, Texas A&M University, College Station, Texas, United States of America; University of Tennessee Health Science Center, United States of America

## Abstract

The STING (stimulator of interferon genes) protein can bind cyclic dinucleotides to activate the production of type I interferons and inflammatory cytokines. The cyclic dinucleotides can be bacterial second messengers c-di-GMP and c-di-AMP, 3’5’-3’5’ cyclic GMP-AMP (3’3’ cGAMP) produced by *Vibrio cholerae* and metazoan second messenger 2’5’-3’5’ Cyclic GMP-AMP (2’3’ cGAMP). Analysis of single nucleotide polymorphism (SNP) data from the 1000 Genome Project revealed that R71H-G230A-R293Q (HAQ) occurs in 20.4%, R232H in 13.7%, G230A-R293Q (AQ) in 5.2%, and R293Q in 1.5% of human population. In the absence of exogenous ligands, the R232H, R293Q and AQ SNPs had only modest effect on the stimulation of IFN-β and NF-κB promoter activities in HEK293T cells, while HAQ had significantly lower intrinsic activity. The decrease was primarily due to the R71H substitution. The SNPs also affected the response to the cyclic dinucleotides. In the presence of c-di-GMP, the R232H variant partially decreased the ability to activate IFN-βsignaling, while it was defective for the response to c-di-AMP and 3’3’ cGAMP. The R293Q dramatically decreased the stimulatory response to all bacterial ligands. Surprisingly, the AQ and HAQ variants maintained partial abilities to activate the IFN-β signaling in the presence of ligands due primarily to the G230A substitution. Biochemical analysis revealed that the recombinant G230A protein could affect the conformation of the C-terminal domain of STING and the binding to c-di-GMP. Comparison of G230A structure with that of WT revealed that the conformation of the lid region that clamps onto the c-di-GMP was significantly altered. These results suggest that hSTING variation can affect innate immune signaling and that the common HAQ haplotype expresses a STING protein with reduced intrinsic signaling activity but retained the ability to response to bacterial cyclic dinucleotides.

## Introduction

The innate immune system is the first line of defense against microbial pathogen infection, including viral and bacterial infection, and tissue damage. It is initiated by germ line-encoded pattern recognition receptors to detect ligands from the pathogen as well as from damage-associated molecular patterns [1]. Ligand recognition by the receptors will lead to production of interferons, proinflammatory chemokines and cytokines, and anti-microbial peptides. A growing numbers of these receptors have been identified, including membrane associated Toll-like receptors (TLRs), cytosolic retinoic-acid inducible gene I (RIG-I)-like receptors, nucleotide-binding-oligomerization-domain (NOD)-like receptors, and double-strand DNA sensors such as DAI, IFI16, AIM2 and DDX41 [1-5].

 STING, also known as MITA, MPYS, ERIS, and TMEM173, was recognized as the signaling adaptor in the innate immune response [6-8], that could detect cytosolic dsDNA through distinct DNA sensors [2-5,9,10]. The C-terminal portion of STING can bind and activate the TBK1 kinase, followed by recruitment and phosphorylation of the transcription factor IRF3, which is essential for triggering the type I interferon response [11]. STING can also directly bind to the bacterial second messenger molecules cyclic diguanylate monophosphate (c-di-GMP) and cyclic diadenylate monophosphate (c-di-AMP) and trigger the innate immune response [12-14]. Recently, it was reported that cyclic GMP-AMP (cGAMP) could also bind to STING and result in activation of IRF3 and β-interferon production [15,16]. Both 3’5’-3’5’ cGAMP (to be designated 3’3’ cGAMP) produced by *Vibrio cholera* [17], and the metazoan secondary messenger cyclic [G(2’,5’)pA(3’5’)] (to be named 2’3’ cGAMP), could activate the innate immune response through STING pathway [18-21]. In addition, STING seems to play an important role in the autoimmune diseases by the inappropriate recognition of self DNA [22].

Crystal structures of human STING (hSTING) complexed with c-di-GMP reveal that the C-terminal portion of STING forms a V-shaped dimer and binds a c-di-GMP molecule at the dimer surface. In addition, the STING/c-di-GMP complex exhibits two distinct conformations (open and closed) in solution [23-27]. Most of the hSTING proteins used for structural determination had substitutions in the C-terminal region of the protein that are located near the ligand binding pocket of hSTING [23-25,27]. This prompted us to examine whether SNPs in hSTING could affect the recognition of cyclic dinucleotides and the innate immune signaling. 

Four major non-synonymous variants of hSTING were found in high frequencies in 1000 Human Genome Project database. Two variants contain single amino acid substitution R232H and R293Q, respectively. The third is a haplotype that consists of two co-segregating substitutions, G230A and R293Q (to be designated AQ). The fourth has three substitutions: R71H, G230A, and R293Q (to be designated HAQ). This observation is similar to the previous report by Jin et al [28] who have identified that HAQ is a loss-of-function hSTING variant from two cohorts of American DNA samples. In this work, we determine whether hSTING SNPs could affect the recognition with exogenous cyclic dinucleotides from bacterium and metazoan. We found that all four major SNPs could recognize the metazoan second messenger 2’3’ cGAMP, while they responded differentially to bacterial cyclic dinucleotides. Furthermore, we determined that the G230A substitution in the HAQ isoform has an altered binding to c-di-GMP relative to the wild-type STING. 

## Materials and Methods

### Sequence and hSTING SNP analysis

SNP data were downloaded in VCF format from the NCBI 1000 Genomes browser [29]. Population code and sample descriptions were obtained from NCBI. The reference genome sequence of human STING was from rs1131769. Haplotype determinations were derived from the downloaded sequence and SNP data using a customized Perl script, available upon request.

### Plasmid constructions

cDNA for hSTING that includes a C-terminal Flag epitope tag was cloned into pCDNA3.1. STING variants were constructed by site-directed mutagenesis using the QuickChange Kit according to the protocol provided by the manufacturer. Sequences of all mutants were confirmed by DNA sequencing using the BigDye Kit (Invitrogen). For recombinant protein expression, residues 149-341 of the hSTING that contained a C-terminal 6×His tag was constructed as described in Shu et al. [23].

### Luciferase reporter assays

HEK293T cells were cultured as previously described in Ranjith-Kumar et al. [30]. The cells were seeded for 24 h in CoStar White 96-well plates prior to transfection with a constant amount of reporter plasmid IFN-β-firefly luciferase (30 ng), or NF-κB-firefly luciferase plasmid (15 ng), phRL-TK-*Renilla* luciferase (2 ng) and varied amounts of WT or mutant hSTING. All transfections were amended with empty pCDNA3.1, as needed, to allow equal amounts of plasmid transfected. The transfection reagent was Lipofectamine 2000 (Invitrogen). After a 24-h incubation, the medium was removed, and the cells were stimulated with cyclic dinucleotides (Biolog) in a solution with 10 μg/ml digitonin at 37°C as described in Woodward et al. [31]. The solution was removed and 200 μl of culture medium was added to each well after a 30-min incubation. The luciferase activity was analyzed using the Dual-Glo Luciferase Reporter Assay (Promega) in the BioTek Synergy2 plate reader. 

### Analysis of phosphorylated IRF3

HEK293T cells were plated on 24-wells plate. Approximately 10 ng of WT or SNPs was transfected into cells using Lipofectamine. After 24h, the cells were stimulated with c-di-GMP (20 μg/ml), c-di-AMP (6 μM), 3’3’cGAMP (6 μM) or 2’3’cGAMP (6 μM) in digitonin permeabilization solution as described in Materials and Methods. The cells were washed with PBS and lysed in the 1X passive lysis buffer (Promega). The lysate was loaded on 4-12% SDS-PAGE and transferred to the PVDF membrane. The blot was first probed with primary antibody rabbit anti-p-IRF3 (Cell Signaling) and followed by HRP-labeled goat anti-rabbit secondary antibody. After stripping, the blot was re-probed with mouse anti-flag monoclonal antibody and goat anti-mouse-HRP secondary antibody to detect the STING expression. The signaling of p-IRF3 that normalized to the loading control (LC) was quantified by Chemidoc software. 

### Western blots

Cells were lysed in a buffer containing 50 mM Tris-HCl, pH 7.5, 150 mM NaCl, 1% nonidet P40, 0.5% sodium deoxycholate and a cocktail of proteinase inhibitors (Roche). The lysate was clarified by centrifugation at 16,000 x g for 10 min. Twenty-five micrograms of the soluble protein were separated by 4-12% SDS-PAGE and then transferred to nitrocellulose membrane. After blocking with 5% BSA in Tris buffered saline (TBS), the membrane was incubated with a mouse anti-Flag monoclonal antibody (Invitrogen) and mouse anti-tubulin antibody (Abcam). After three washes with TBS amended with 0.1 % Tween-20, the membrane was probed with HRP-labeled Goat-anti-mouse IgG or Goat-anti-rabbit IgG, and the signal was detected with ECL prime detection reagent (GE Healthcare). The tubulin was detected as the sample loading control. 

### Expression and purification of the C-terminal domain of hSTING

Residues 149-341 of the hSTING that contained a C-terminal 6×His tag was expressed in *E. coli* BL21 (DE3) as described in Shu et al. [23]. The protein was purified by nickel affinity chromatography followed by gel-filtration chromatography. The purified protein was stored at -80°C in the buffer containing 20 mM Tris-HCl (pH 7.5) and 150 mM NaCl. hSTING containing the G230A substitution was made by site-directed mutagenesis and the protein purified and stored using the same protocol as for WT hSTING. 

### Isothermal titration calorimetry (ITC)

Dissociation constants and the thermodynamic parameters for hSTING and c-di-GMP binding were measured using a MicroCal ITC200 calorimeter (Northampton, MA). Titrations of c-di-GMP used a 1 mM solution and were injected as 2 μl aliquots into a 0.2 mM solution of WT or G230A hSTING at 25°C. The reference offset was at 10 μcal/s, the syringe stirring speed was 1000 rpm, a pre-injection delay was set for 180 s, and the data were recorded at 5 s intervals. Equilibrium association constant, binding stoichiometry (N) and enthalpy (ΔH) were determined by fitting the data based on a single set of identical sites model using Origin7 (Microcal).

### Differential scanning fluorimetry (DSF)

The thermal denaturation profile of recombinant hSTING proteins was determined using a Stratagene MX3005P machine. The samples were prepared in 25 μl reactions that contained a 10 Χ final concentration of SYPRO orange (Invitrogen), 20 μM WT hSTING or G230A protein, and a molar excess of c-di-GMP relative to hSTING proteins, in a buffer containing 50 mM Tris-HCl (pH 7.5) and 150 mM NaCl. All samples were heated from 25 to 75°C with a ramp rate of 0.5°C/min. The fluorescence intensity data were analyzed using programs from GraphPad Prism.

### Analysis of STING Structures

Crystal structures of STING were downloaded from the Protein Data Bank (www.rcsb.org). The Chimera software from the University of California San Francisco (http://www.cgl.ucsf.edu/chimera/) was used to superposition the structures and to prepare figures. 

## Results

### STING isoforms in human populations

Although growing evidences suggest that the genetic variation of innate immune sensors could affect the susceptibility of human diseases [32,33], the linkage between single nucleotide polymorphisms in human STING and diseases remains to be established. We sought to determine the spectrum, potential origins, and extant distribution of hSTING isoforms in the human population and whether the SNPs will affect STING’s ability to activate signal transduction. We examined the haplotypes of 1,090 individuals whose genomes have been sequenced as part of the 1000 Genomes project [29]. 76 bi-allelic SNPs are observed in the 7,231-bp STING gene, including 7 in the 5’ untranslated regions (UTR), 26 in the coding sequence, 38 in introns, and 5 in the 3′ UTR, corresponding to rates per 1 kb of 6.64, 22.81, 8.83, and 6.82, respectively. Thus, SNPs occur about three times more often in the STING coding sequence when compared to the UTRs or the introns. Moreover, 20 of the 26 SNPs in the coding sequence are non-synonymous substitutions, suggesting a lack of purifying selection and possibly positive selection for hSTING variants. 

### Human STING non-synonymous SNPs analysis

**Figure 1:**
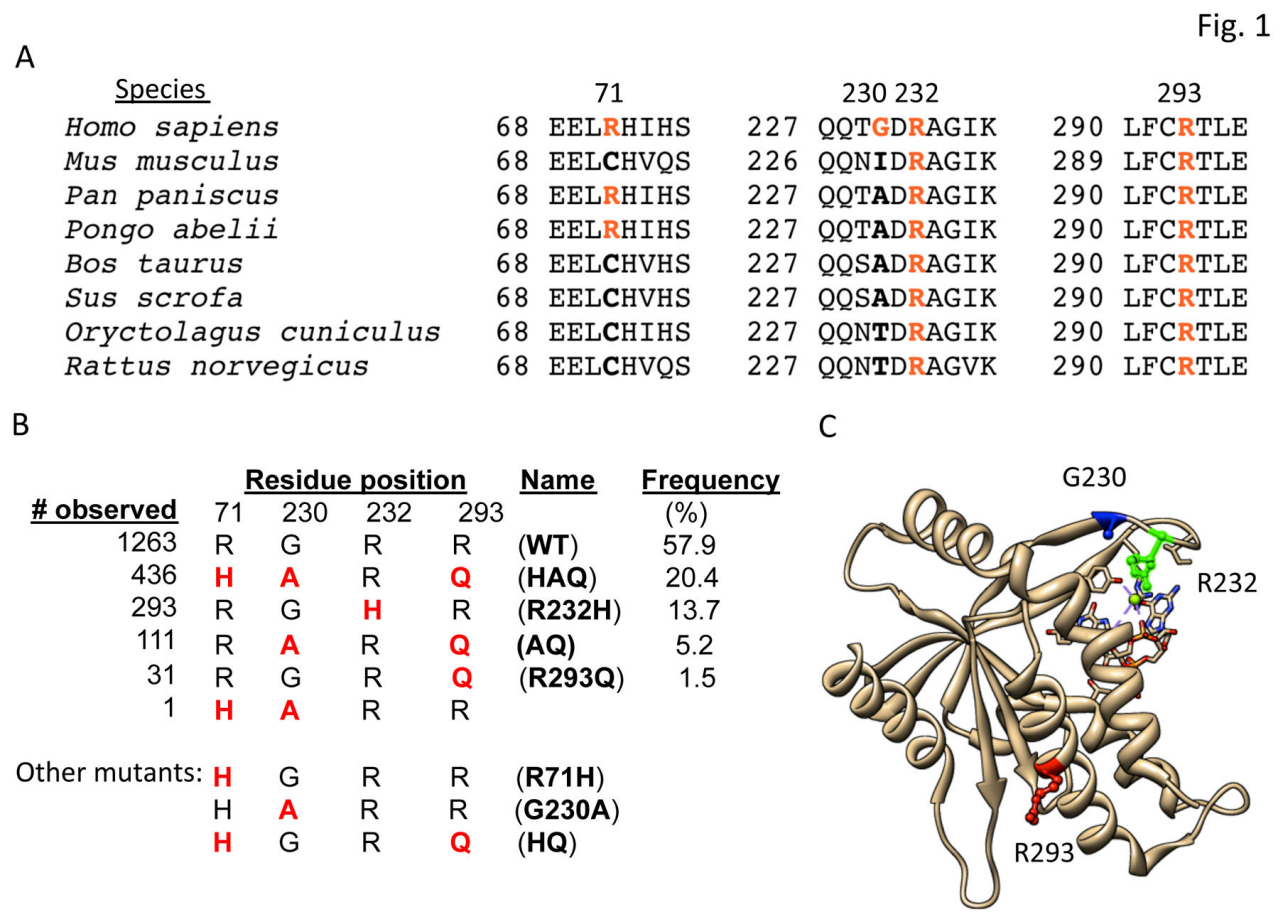
Analysis of single nucleotide polymorphisms in the coding region of hSTING. (A) Sequence alignment of STING from different species. The four variations in the coding region of hSTING, R71, G230, R232 and R293 are shown in bold and colored in red. The evolutional conservation of R232 and R293 among the species is highlighted. The residue numbers denote those in hSTING. (B) Allele frequency of hSTING. The frequencies derived from 1000 Genome Project database are in the left-most column and the amino acid variations are colored in red. The name of hSTING SNPs and mutants studied in this work are shown in bold and the frequency is calculated in the right-most column. (C) Localization of G230, R232 and R293 in the crystal structure of hSTING in complex with c-di-GMP. A monomer of the hSTING bound to c-di-GMP is shown to allow ease of identification of the residues. Residues G230, R232 and R293 are respectively, colored blue, green and red.

There are four major hSTING isoforms in the 1000 Genome Project: R232H, R293Q, G230A-R293Q (AQ) and R71H-G230A-R293Q (HAQ) (Figure 1B). The R232H and HAQ haplotypes are present in 13.7% and 20.4%, respectively, of the human population, while AQ and R293Q are 5.2% and 1.5%, respectively (Figure 1B). Residue R71 is located in the transmembrane domain of hSTING while residues R232 and G230 are located in the loop structure which was predicted to form the c-di-GMP binding pocket of hSTING (Figure 1C).

Notably, several of the hSTING proteins previously characterized for activity and used for structural analysis contained the R232H SNP [23-25]. The R232H allele has a thymidine at nucleotide position 4425 (numbering relative to the gene sequence). However, a cytosine is the most common nucleotide at position 4425, and this encodes R232. Thus hSTING with R232 will be considered as WT, and hSTING with H232 (corresponding to dbSNP entry rs1131769) will be considered as SNP-derived isoform.

With only one exception in the 1000 genome data set, both the R71H and G230A substitutions are independently associated with R293Q. The R293Q substitution is thus present in the three major variants (R293Q, AQ and HAQ) and accounts for 27% of the variation in hSTING. Alignment of the STING sequences reveals that R232 and R293 are highly conserved among diverse species (Figure 1A). 

### Phylogeny and frequencies of SNPs in human populations

**Figure 2:**
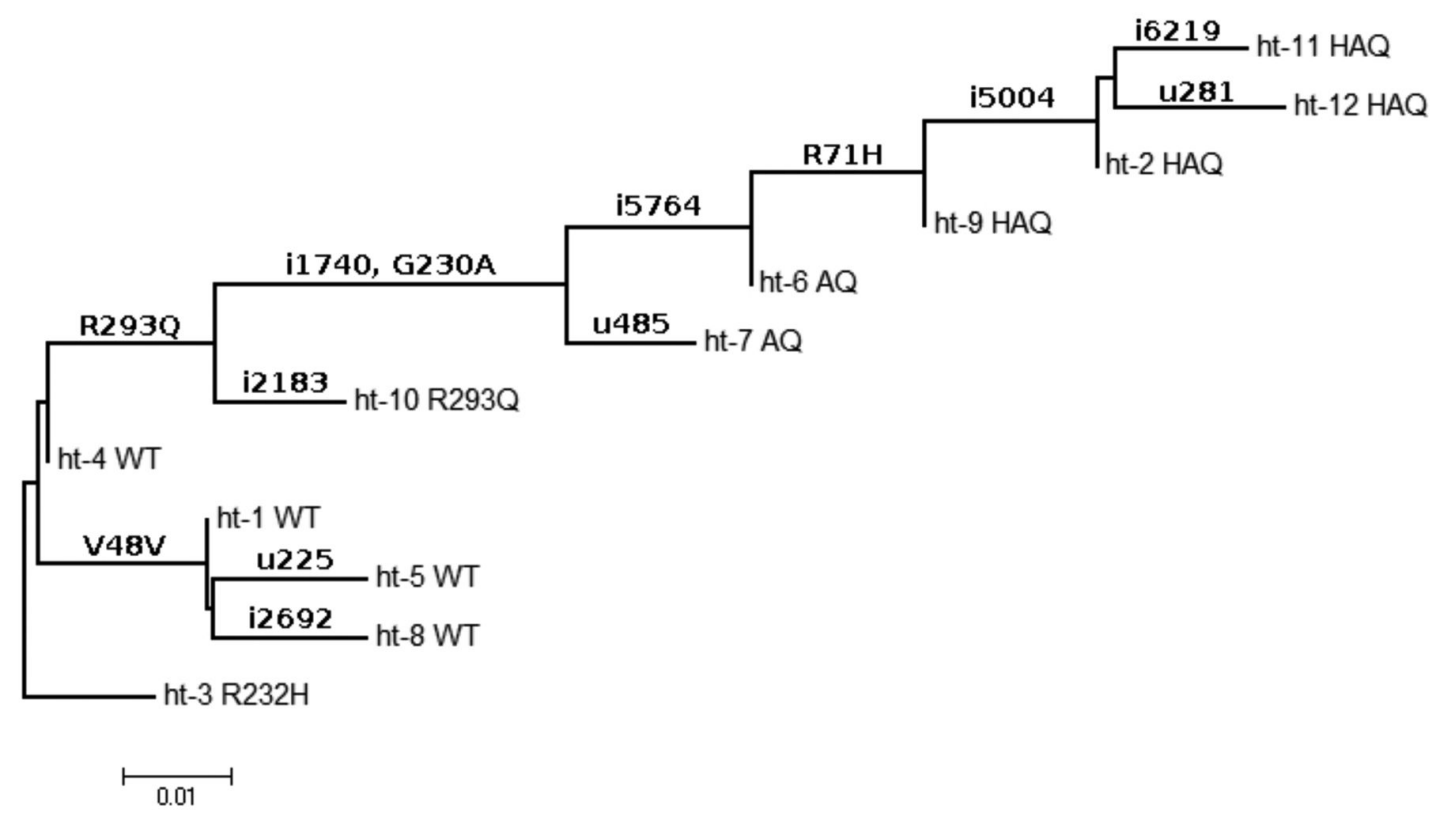
Phylogeny of hSTING haplotypes in human populations. A possible evolutionary history of the 12 major human STING haplotypes (ht-1 to ht-12) was inferred by the minimum evolution model implemented in MEGA5 with default settings [45]. Input sequences were the haplotype-specific nucleotides in the 76 SNP positions observed in the STING gene. The SNP positions are labeled above branches and numbered relative to the gene sequence and locations. In the latter, “I” denotes location in intron and “u” for the 5’UTR. SNPs in coding regions are labeled in the conventional “reference residue/amino acid position/alternative residue” style. Tree leaves are labeled according to the encoded protein isoforms as in Figure 1B.

The 12 most common haplotype sequences, named ht1 through ht12 in order of abundance, can be ordered in a phylogenetic pattern of descent as shown in Figure 2. The predominant isoform (WT hSTING) is derived from ht-1, ht-4, ht-5, and ht-8 (a total of 1169 occurrences among the 2180 in 1000 Genomes Project alleles); R232H derives from ht-3; R293Q (26 occurrences) derives from ht-10; AQ (93 occurrences) derives from ht-6 and ht-7; and HAQ (420 occurrences) derives from ht-2, ht-9, ht-11, and ht-12 (Figure 2). A likely succession of changes was R293Q, then G230A, and then R71H, as supported by the observation that all 438 instances of R71H occur in the G230A context; only one out of 550 G230A instances is not associated with R293Q; and there are 31 instances of R293Q on its own.

The R293Q and AQ isoforms occur predominantly within the African subpopulation, while HAQ is much higher in the Asian and Hispanic American subpopulation than in the African and European subpopulations (Table 1). Strikingly, the R232H variant is the only haplotype with a distribution that does not significantly deviate from the overall sampling of the populations. Common SNPs in the non-coding portions of the genes suggest haplotype origins predating the radiation of ethnicities (Figure 2). The biased distributions of the haplotypes reflect specific selection for particular protein isoforms in geographically separated populations. 

**Table 1:** hSTING haplotype distribution among human subpopulations.

			**AFR**			**ASN**			**EUR**					**HIS**		
***ht***	***count***	***isoform***	*ASW*	*LWK*	*YRI*	*CHB*	*CHS*	*JPT*	*CEU*	*FIN*	*GBR*	*IBS*	*TSI*	*CLM*	*MXL*	*PUR*
All	2180		5.5	8.9	7.98	8.9	9.17	8.17	7.8	8.53	8.07	1.28	8.99	5.5	6.06	5.05
			**22.39**			**26.24**			**34.68**					**16.61**		
ht-2	352	HARQ	1.14	0.85	0	15.34	18.75	16.76	4.55	6.82	7.39	1.7	9.38	5.97	6.25	5.11
			**1.99**			50.85*			**29.83**					**17.33**		
ht-12	16	HARQ	0	0	0	31.25	18.75	50	0	0	0	0	0	0	0	0
			**0**			100*			**0**					**0**		
ht-11	21	HARQ	9.52	0	0	0	0	0	0	0	0	0	0	28.57	61.9	0
			**9.52**			**0**			**0**					90.48*		
ht-9	31	HARQ	0	3.23	3.23	25.81	38.71	22.58	3.23	0	3.23	0	0	0	0	0
			**6.45**			87.1*			**6.45**					**0**		
ht-6	56	RARQ	26.79	39.29	28.57	0	0	0	0	0	0	0	0	5.36	0	0
			94.64*			**0**			**0**					**5.36**		
ht-7	37	RARQ	24.32	24.32	37.84	0	0	0	0	0	0	2.7	0	0	2.7	8.11
			86.49*			**0**			**2.7**					**10.81**		
ht-10	26	RGRQ	0	73.08	26.92	0	0	0	0	0	0	0	0	0	0	0
			100*			**0**			**0**					**0**		
ht-3	286	RGHR	4.2	5.59	6.99	5.24	8.39	7.34	9.44	7.69	11.89	1.4	9.79	7.34	4.9	9.79
			**16.78**			**20.98**			**40.21**					**22.03**		
ht-4	222	RGRR	11.71	31.08	28.83	8.11	5.41	4.5	0	0.9	0.9	0	0.9	3.15	1.8	2.7
			71.62*			**18.02**			**2.7**					**7.66**		
ht-1	823	RGRR	2.19	0.73	0.49	10.09	7.9	8.14	12.88	13.49	11.66	1.46	12.88	5.83	7.41	4.62
			**3.4**			**26.12**			52.37*					**17.86**		
ht-5	91	RGRR	5.49	1.1	3.3	0	0	0	15.38	18.68	13.19	2.2	16.48	8.79	6.59	8.79
			**9.89**			**0**			65.93*					**24.18**		
ht-8	33	RGRR	9.09	0	0	0	0	0	12.12	27.27	3.03	6.06	15.15	6.06	9.09	12.12
			**9.09**			**0**			63.64*					**27.27**		

Haplotype occurrence counts (column 2) and percentages relative to sampled subpopulations (columns 3-16). Protein isoforms are indicated in column three with labels as explained in the legend to Figure 1. HARQ denotes H71-A230-R232-Q293. Strongly ethnicity-specific haplotype occurrences are denoted with asterisks.

### Effects of SNPs on hSTING expression and activity

**Figure 3:**
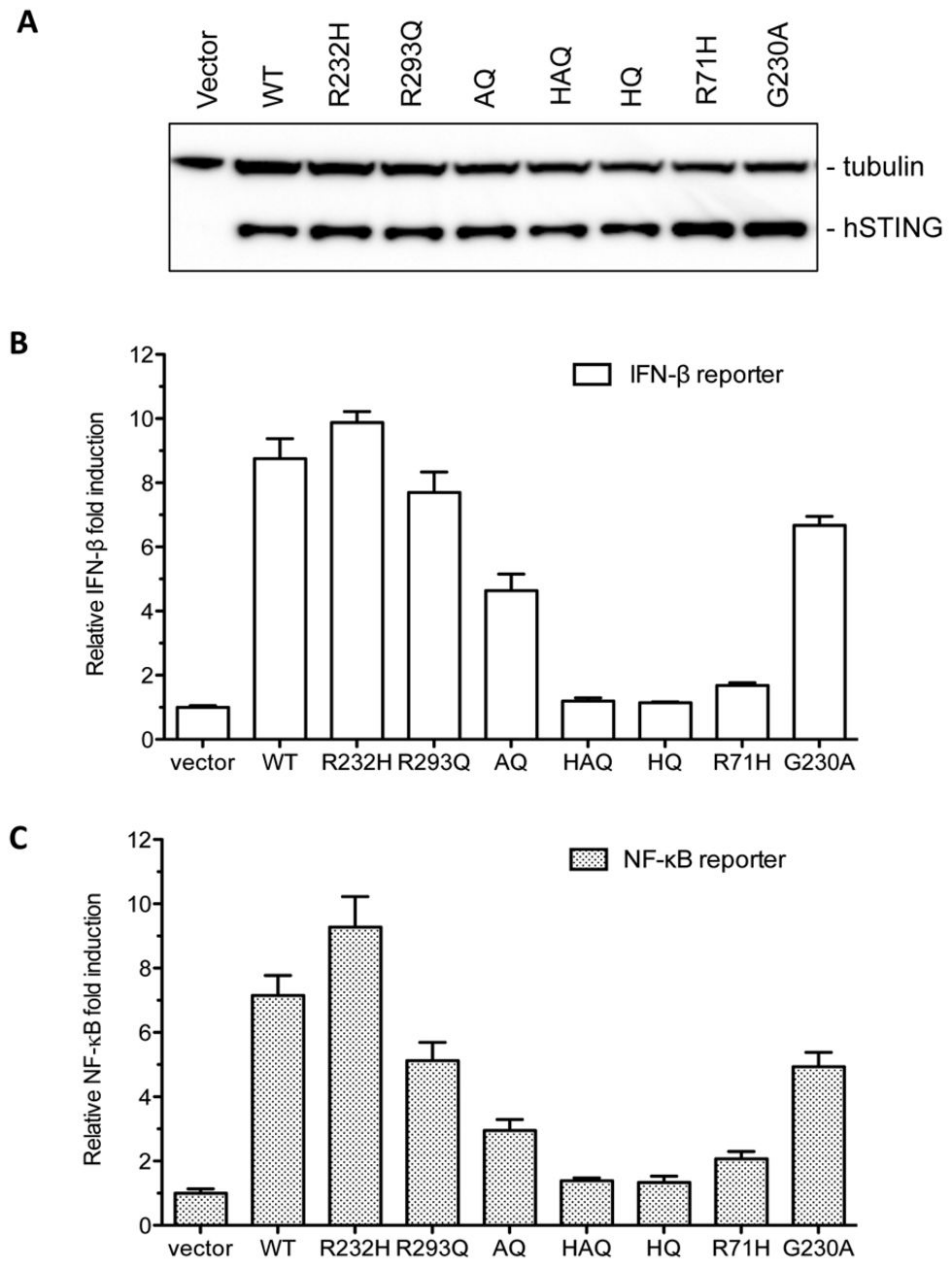
Expression and un-induced activities of WT hSTING and SNPs. (A) Western blot analysis the expression level of hSTING SNPs and additional mutants in HEK293T cells transfected with equal amount of WT or mutant plasmid for 30h. Approximately equal amount of cell lysate was separated by SDS-PAGE followed by detecting the Flag-tagged STING with mouse anti-flag antibody. Tubulin was used as a sample loading control. (B) IFN-β luciferase reporter assay. Equal amount of each plasmid was transfected into HEK293T cells together with constant amount of IFN-β Firefly luciferase reporter and *Renilla* luciferase. The ratio of Firefly to *Renilla* luciferases was plotted as relative IFN-β fold induction. The vector is used as negative control and all the data was normalized to vector control. (C) NF-κB reporter assay. The ratio of Firefly luciferase to *Renilla* luciferase was plotted as relative fold of NF-κB induction.

A single amino acid substitution in STING has been reported to affect protein stability and the ability to activate IFN-β production in mouse and human cells [13,34]. We seek to examine the expression level of hSTING variants in HEK293T cells, which do not express detectable endogenous hSTING but are competent for signaling in response to transiently expressed STING and exogenously provided ligands [12,13]. Western blot analysis was performed to detect the Flag epitope added to C-terminus of hSTING. The four major SNPs, R232H, R293Q, AQ and HAQ, and several additional mutants R71H, G230A, and R71H-R293Q (HQ), which were constructed to elucidate the specific effects of the multiple substitutions in the haplotypes, were expressed at similar levels to that of WT hSTING (Figure 3A), indicating that these variants did not obviously alter hSTING expression and/or stability in HEK293T cells. 

To determine whether the hSTING variants could affect the activation of innate immune responses, HEK293T cells were transfected with IFN-β firefly luciferase reporter together with either WT or mutants hSTING. *Renilla* luciferase, expressed under a constitutive thymidine kinase promoter from the phRL-TK-*Renilla* luciferase plasmid, served as a transfection control. The ratio of firefly luciferase to the *Renilla* luciferase was plotted to provide fold induction. Substitutions G230A and R293Q decreased IFN-β activation by a modest 20% relative to WT, while the AQ mutant decreased activation by 30-40%, presumably due to the additive effects of the G230A and R293Q substitutions. The R232H SNP slightly enhanced the IFN-β readout (Figure 3B). Strikingly, three mutants, R71H, HQ and HAQ, dramatically decreased the activation of IFN-β promoter by more than 80% relative to the WT (Figure 3B). Of the three substitutions in HAQ haplotype, the R71H substitution is primarily responsible for the reduction of signaling from the IFN-β reporter.

hSTING has been reported to activate stress response through the NF-κB transcription factor [35]. When tested for the effects on firefly luciferase expression through the NF-κB promoter element, the results closely parallel those from the IFN-β promoter (Figure 3C). These results confirm that the major hSTING variants found in the human population have dramatically distinct level of ligand-independent signal transduction. 

### Effects of hSTING SNPs on cyclic dinucleotides-mediated innate immune response

**Figure 4:**
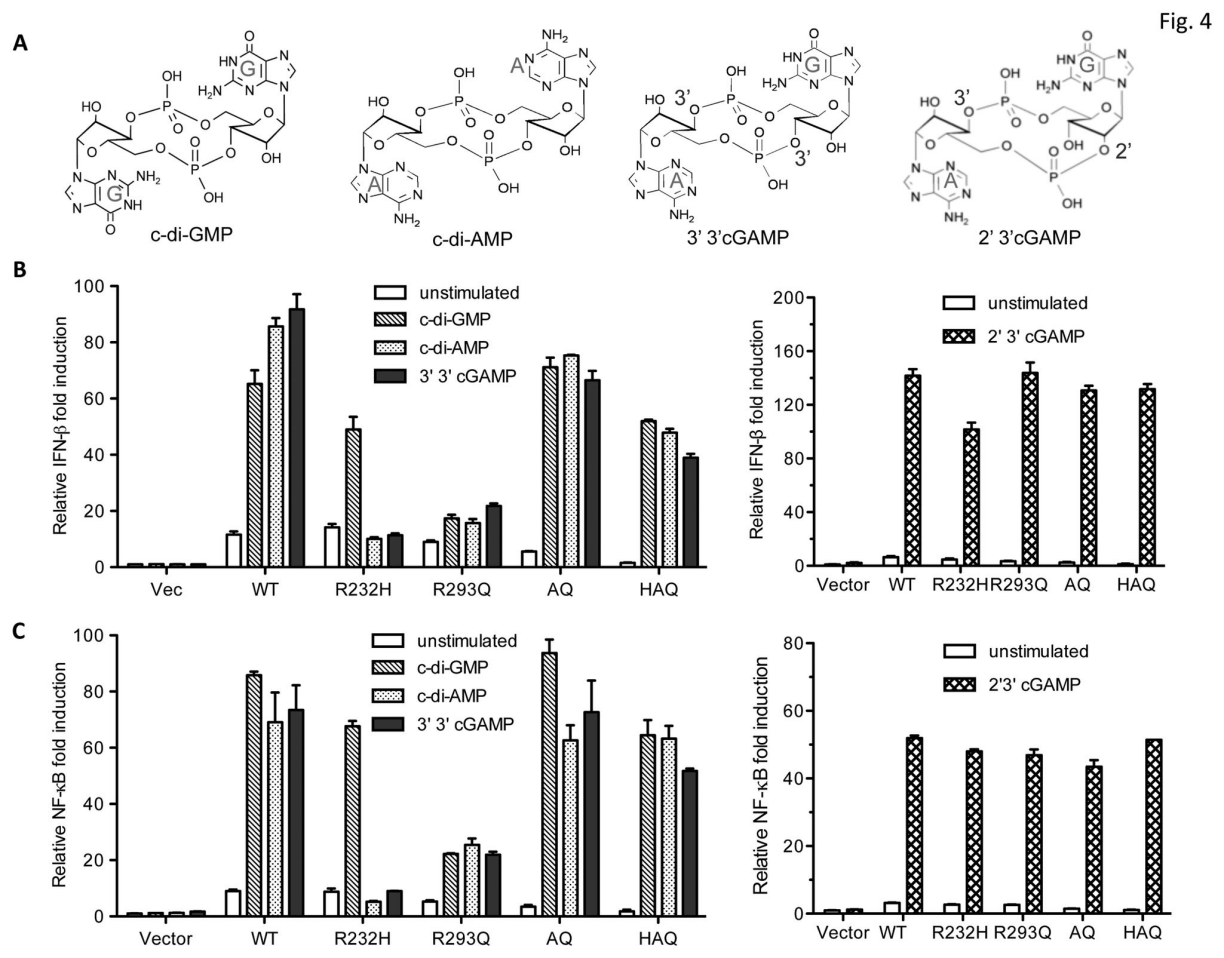
Effects of cyclic dinucleotides-mediated activation of IFN-β and NF-κB promoter activity by WT hSTING and the SNPs. (A) Structures of cyclic di-GMP, cyclic di-AMP, 3’3’cGAMP and 2’3’cGMP. (B) Assay with the IFN-β reporter in transiently-transfected HEK293T cells. The cells were first transfected with 2 ng of either WT hSTING or mutants together with IFN-β firefly luciferase and *Renilla* luciferase reporters by Lipofectamine 2000. After 24 h post-transfection, the cells were treated with digitonin permeabilization solution with or without cyclic dinucleotides for 30 min at 37°C incubator. The permeabilization solution was removed and replaced with culture medium. The luciferase activity was read after 6 h incubation and the data was plotted with relative IFN-β fold induction that normalized to vector control. The bars denote the cells were, respectively, mock-treated or treated with c-di-GMP, c-di-AMP, 3’3’cGAMP or 2’3cGAMP. (C) Effects of cyclic dinucletides on hSTING activity with NF-κB reporter. The cells were transfected with WT hSTING or mutants together with NF-κB reporter luciferase.

To address whether the hSTING variants could affect ligand-dependent signaling, cells expressing the homozygous SNPs were tested for the activation of IFN-β promoter activity in the presence of bacterial ligands c-di-GMP, c-di-AMP, 3’3’cGAMP and metazoan ligand 2’3’cGAMP (Figure 4A). C-di-GMP increased IFN-β signaling from WT hSTING to 65-fold relative to the level without exogenous ligands (10-fold; Figure 4B). SNP R232H decreased c-di-GMP-induced response by only 30-40%, while it was dramatically defective for the response to c-di-AMP and 3’3’cGAMP. R293Q reduced the ability to respond to all bacterial ligands by 60-80%. 

Intriguingly, AQ and HAQ haplotype were largely restored for signaling in response to all ligands (Figure 4B), despite lower IFN-β signaling in the absence of the ligand. C-di-GMP-dependent IFN-β signaling by HAQ was restored to 52-fold, ca. to 80% of WT level (Figure 4B). Similar restoration by HAQ was observed for c-di-AMP (49-fold vs 72-fold of WT) and 3’3’ cGAMP (35-fold vs 48-fold of WT). The AQ variant also had similar response to c-di-GMP, c-di-AMP and 3’3’cGAMP relative to WT hSTING (Figure 4B; Table 2). Comparable results from the NF-κB promoter-reporter were also observed for AQ and HAQ variants (Figure 4C). The 2’3’cGAMP contains a mixed phosphodiester linkage that was recently demonstrated to be the natural product of cyclic GMP-AMP synthase [18-21]. Despite the SNPs had different response to the bacterial cyclic dinucleotides, all the hSTING SNPs could recognize 2’3’ cGAMP and activated either the IFN-β or the NF-kB reporters to at least 100-fold above the background. While R232H had a robust response to 2’3’ cGAMP, it had a modest decrease in the activation of IFN-β reporter activity that is statistically significant when compared to results from the WT STING (p < 0.05) (Figure 4C). These results are consistent with those of Zhang et al. [21]. However, R232H did not have a statistically significant difference from WT with the NF-kB reporter (Figure 4C). 

**Table 2:** Summary of the effects of hSTING SNPs on the activation of IFN-β reporter activity in the presence of bacterial cyclic dinucleotides.

	**Unstimul.**	**+c-di-GMP**			**Unstimul.**	**+c-di-AMP**			**Unstimul.**	**3'3'cGAMP**		
	Mean±SD	Mean±SD	Ratio	*P* value	Mean±SD	Mean±SD	Ratio	*P* value	Mean±SD	Mean±SD	Ratio	*P* value
Vec.	1.0 ± 0.1	1.5 ± 0.2	1.5		1.0 ± 0.1	1.1 ± 0.2	1.1		1.0 ± 0.1	1.3 ± 0.1	1.3	
WT	6.9 ± 0.4	106.9 ± 5.8	15.4		4.5 ± 0.3	72.1 ± 3.8	16.1		6.2 ± 0.4	48.2 ± 1.8	7.8	
R232H	9.6 ± 0.9	69.6 ± 5.1	7.2	0.017	5.0 ± 0.3	4.4 ± 0.2	0.9	<0.001	5.2 ± 0.5	5.7 ± 0.2	1.1	<0.001
R293Q	6.3 ± 0.3	20.2 ± 0.8	3.2	<0.001	4.7 ± 0.2	9.7 ± 0.8	2.1	<0.001	4.8 ± 0.5	14.4 ± 1.1	2.9	<0.001
AQ	6.5 ± 0.3	90.6 ± 3.7	13.9	0.127	5.4 ± 0.1	48.0 ± 5.1	8.9	0.019	2.7 ± 0.4	45.8 ± 1.1	17.1	0.51
HAQ	1.3 ± 0.2	84.5 ± 10.3	63.4	0.196	1.1 ± 0.2	49.2 ± 1.8	44.7	0.005	1.1 ± 0.1	34.9 ± 1.3	30.9	0.07
HQ	1.1 ± 0.1	3.5 ± 0.1	3.1	<0.001	1.1 ± 0.0	4.3 ± 0.2	3.8	<0.001	1.2 ± 0.1	5.4 ± 0.4	4.4	<0.001
G230A	3.1 ± 0.6	130.0 ± 2.9	41.7	0.045	4.6 ± 0.2	96.1 ± 3.7	20.9	0.01	6.5 ± 0.3	73.9 ± 5.2	11.5	<0.001
R71H	1.4 ± 0.2	37.0 ± 2.5	26.5	<0.001	1.4 ± 0.1	43.1 ± 3.2	31.7	0.004	1.8 ± 0.3	46.3 ± 2.7	25.9	0.08

*All *p* values were calculated relative to the WT hSTING for each treatment.

**Figure 5:**
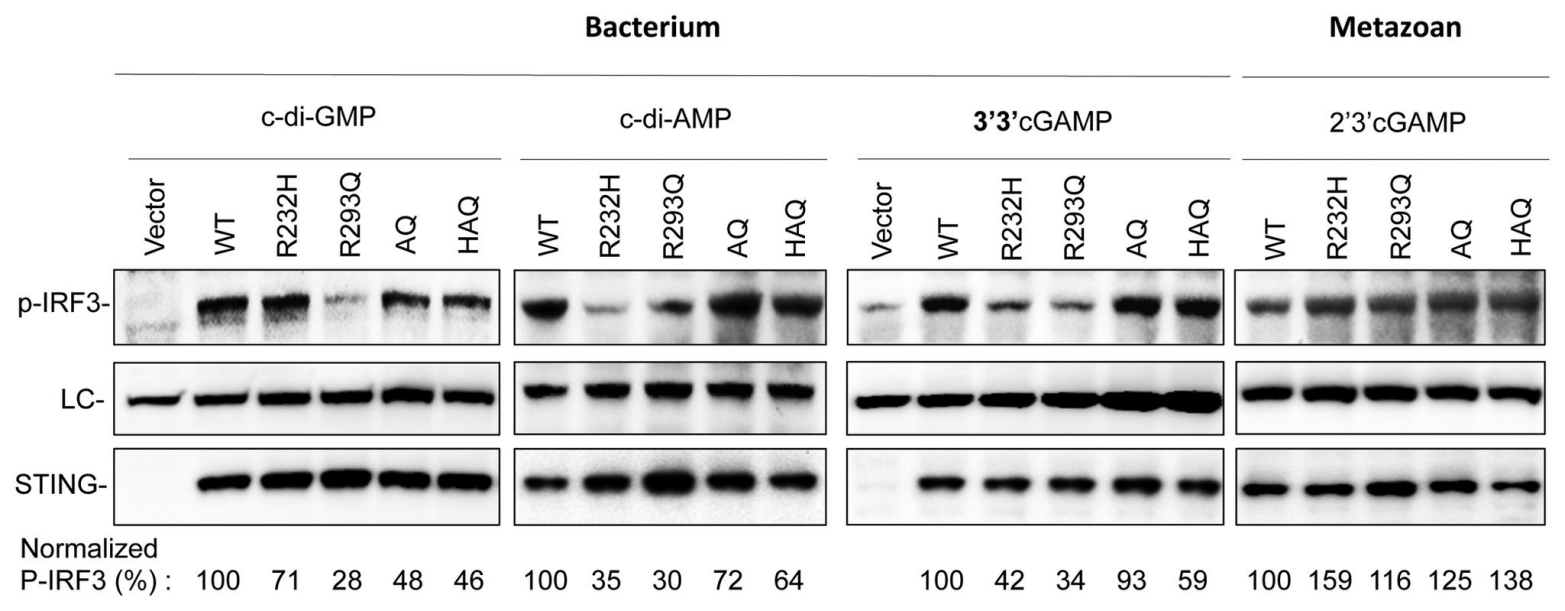
Western blot analysis of p-IRF3 level in response to cyclic dinucleotides. 293T cells transiently transfected to express STING or the SNPs as described in the Methods were harvested and the lysates were probed with antibody to detect p-IRF3 and STING. The loading control (LC) was a non-specific band that cross-reacted with the p-IRF3 antibody. The ratio of the signal present in the p-IRF3 divided by the signal in the LC for the WT STING was normalized to 100%.

To confirm that hSTING variants could differentially recognize the cyclic dinucleotides, we evaluated the amount of phosphorylated IRF3 (p-IRF3) by Western blot. Phosphorylation of IRF3 is required for STING-dependent type I interferon response [11]. In the presence of c-di-GMP, cells expressing R232H isoform had ~70% of the p-IRF3 as the WT. In the presence of c-di-AMP or 3’3’cGAMP, the amount of p-IRF3 was at 30-40% of WT (Figure 5). Despite the low level of p-IRF3 for R293Q stimulated by bacterial cyclic dinucleotides, the AQ and HAQ variants could restore the p-IRF3 level from 50 to 90% of WT level. Intriguingly, all the hSTING SNPs exhibited levels of p-IRF3 comparable to that of WT when stimulated with 2’3’cGAMP (Figure 5). The levels of p-IRF3 for the SNPs are consistent with those from the IFN-β and NF-κB reporter assays. Taken together, these results indicate that human STING variants can only modulate the innate immune response to cyclic dinucleotides derived from bacterium but not from human. 

### G230A substitution is primarily responsible for AQ and HAQ to rescue IFN-β signaling stimulated with c-di-GMP

**Figure 6:**
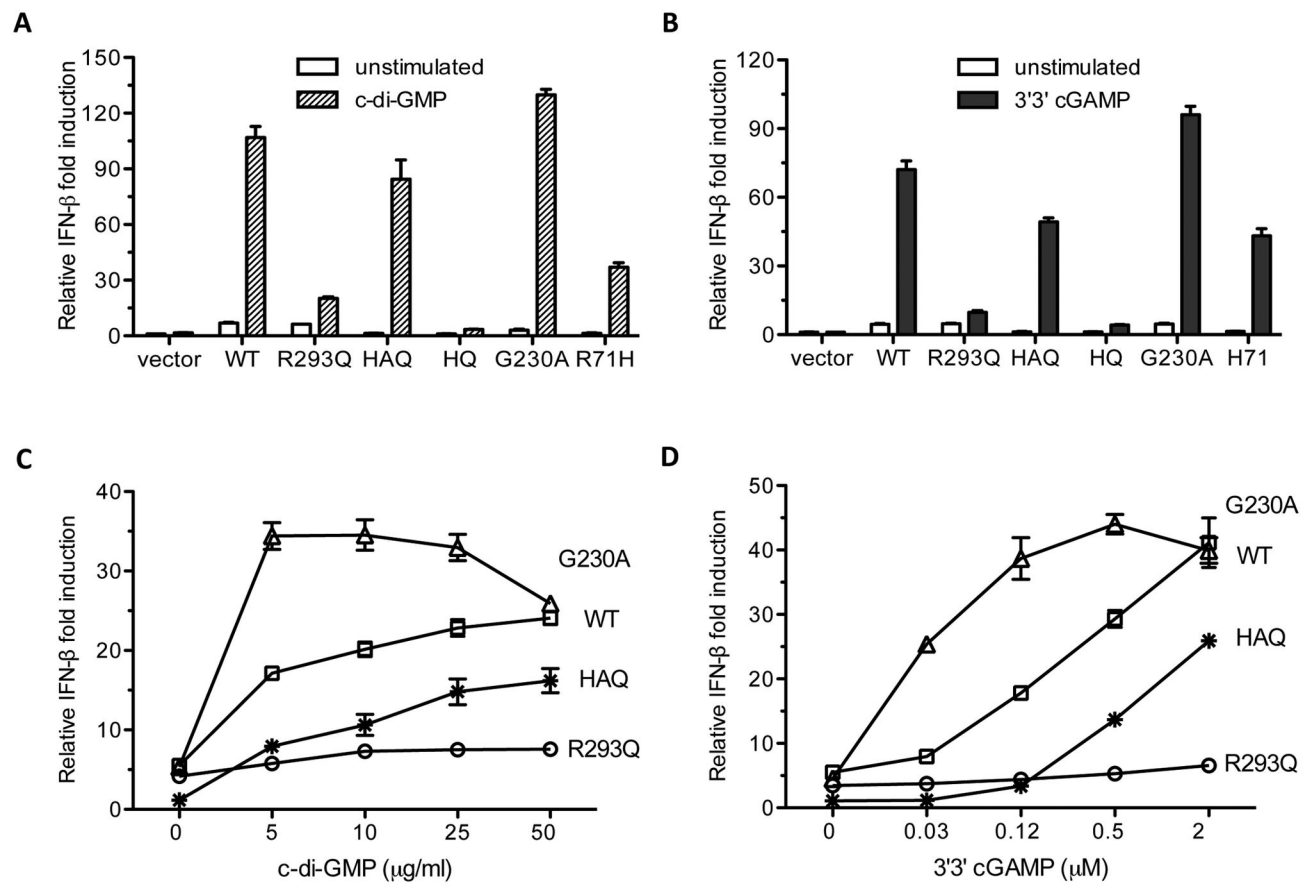
Mutational analysis of substitution in HAQ variant on c-di-GMP or 3’3’cGAMP-dependent IFN-β signaling. (A) Effects of substitutions on c-di-GMP-mediated IFN-β signaling. About 2 ng of each plasmid was transfected into 293T cells for 24h and then the cells were stimulated with 20 μg/ml of c-di-GMP. The ratio of IFN-β signaling stimulated with c-di-GMP via unstimulated was plotted as relative IFN-β fold induction. (B) Effects of substitutions on 3’3’cGAMP-mediated IFN-β signaling. (C) Effects of c-di-GMP concentration on the stimulation of IFN-β activity by hSTING and mutants. The cells were transfected with 2 ng of WT hSTING or mutant plasmid together with IFN-β reporter and further stimulated with different concentration of c-di-GMP at 24h posttransfection. (D) Effects of 3’3’cGAMP concentration on the stimulation of IFN-β signaling.

We postulate that the G230A substitution shared by the AQ and the HAQ haplotypes was responsible for the improved ability to respond to the cyclic dinucleotides. Relative to WT hSTING, G230A hSTING was found to respond better to c-di-GMP (130-fold versus 107-fold), to c-di-AMP (96-fold versus 72-fold) and to 3’3’cGAMP (74-fold versus 48-fold) (Figure 6A and 6B; Table 2). We also observed that the HAQ isoform required the G230A substitution in order to respond to both c-di-GMP and to 3’3’cGAMP, since the HQ isoform was completely nonresponsive to either ligands (Figure 6A and 6B). The results from NF-κB signaling were comparable to those from the IFN-β reporter (Figure S1A and S1B). Finally, the amount of p-IRF3 were produced by the STING variants were consistent with those from the reporter assay (Figure S1C). These results demonstrate that G230A substitution in the AQ and HAQ haplotype was responsible for the ability to respond to bacterial cyclic dinucleotides.

Next, we examined how increasing concentrations of c-di-GMP and 3’3’cGAMP could affect IFN-β signaling. The G230A hSTING signaled to a greater level than that of WT hSTING at lower concentrations of c-di-GMP and 3’3’cGAMP tested (Figure 6C and 6D). In these experiments, a mutant known to be defective for signaling, R293Q, was severely defective for signaling at all concentrations of c-di-GMP and 3’3’cGAMP tested. Signaling by G230A reached maximal levels in cells transfected with 5 μg/ml of c-di-GMP while WT and HAQ continued to increase with up to 50 μg/ml of c-di-GMP (Figure 6C). In the presence of 3’3’cGAMP, G230A could be stimulated at 0.03 μM, while WT and the HAQ variant had no response (Figure 6D). These results suggest that G230A can recognize cyclic dinucleotides in a manner distinguishable from WT hSTING. 

### Effects of concentration of hSTING SNPs on c-di-GMP-mediated signaling

**Figure 7:**
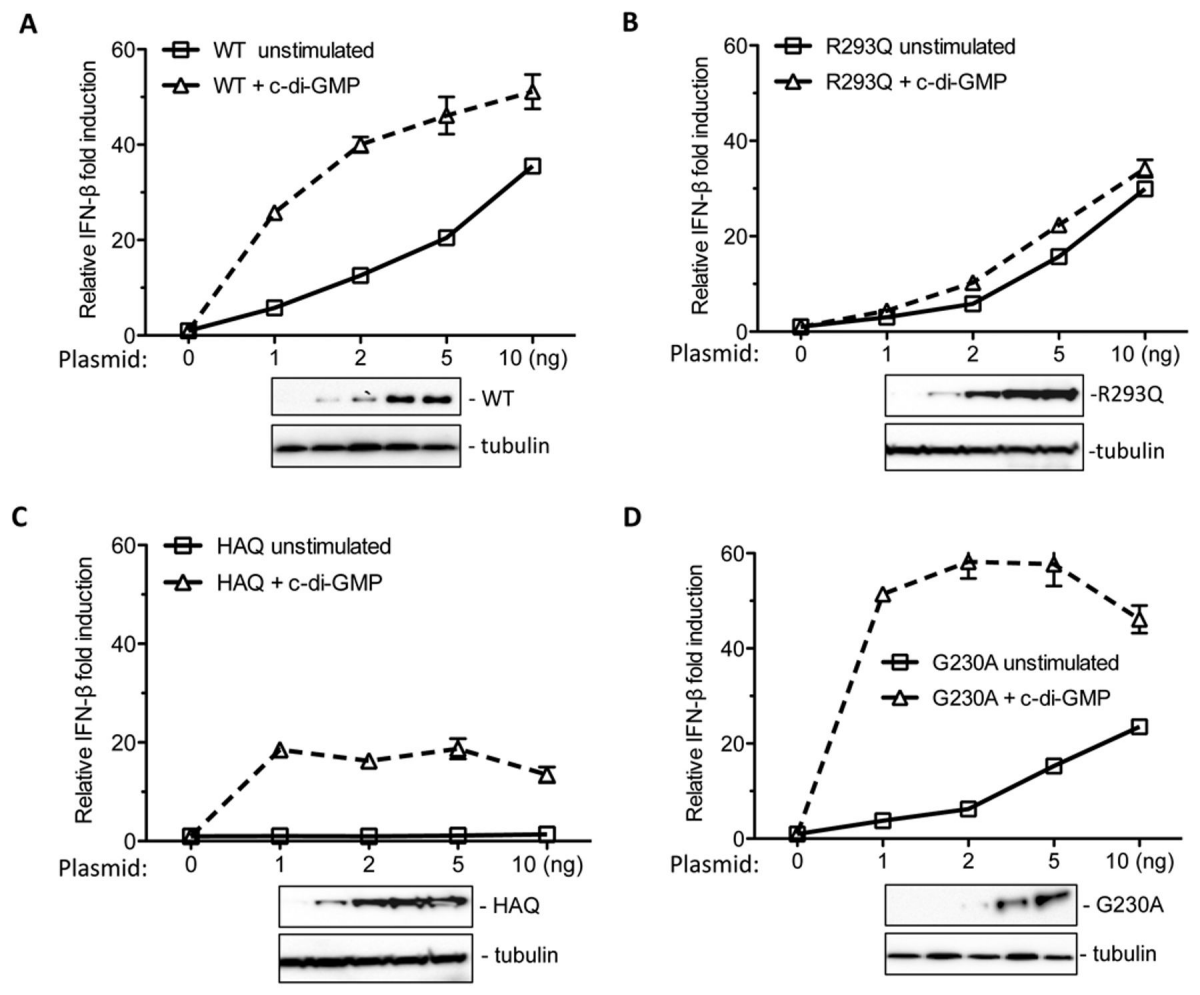
Effects of hSTING expression and c-di-GMP ligand on the activation of IFN-β promoter activity. (A) Effects of WT hSTING concentration on the stimulation of IFN-β activity. HEK293T cells were transfected with indicated concentration of plasmid followed by the addition of c-di-GMP. The solid line denotes unstimulated samples and dash line represents further stimulation by c-di-GMP. Western blot analysis of WT hSTING expression in cells was shown below. The tubulin was probed as the sample loading control. (B) Effects of R293Q concentration on IFN-β activity with or without c-di-GMP. (C) Effects of HAQ concentration on IFN-β activity in the absence or presence of c-di-GMP. (D) Effects of G230A concentration on IFN-β activity.

Sensing c-di-GMP ligand depends on the level of STING protein expressed in HEK293T cells [13]. Thus, we examined the effects of hSTING concentration on c-di-GMP-dependent signaling. Plasmid encoding the WT or mutants hSTING were transfected into cells at 1, 2, 5 and 10 ng. Western blot analyses confirmed that the accumulation of the WT or mutant STING increased with the increasing amount of plasmid transfected (Figure 7). Increasing hSTING expression enhanced c-di-GMP signaling from the IFN-β promoter, by 3 to 5-fold relative to basal levels (Figure 7A). As a control, the R293Q variant was defective for response to c-di-GMP at all the concentration tested (Figure 7B). The HAQ hSTING restored the response to about 80% of WT level when transfected with 1 ng plasmid (Figure 7C). Unlike WT hSTING, however, higher HAQ expression did not affect the level of signaling (Figure 7C). For G230A mutant, it strongly enhanced IFN-β signaling at even lower concentrations of plasmids (Figure 7D). At 1 ng of plasmid expressing G230A, the IFN-β readout was increased by 50-fold in the presence of c-di-GMP, twice the level of the response by the WT hSTING (25-fold). These results confirm that G230A is more responsive to c-di-GMP than the WT hSTING and likely contribute to the ability of the HAQ STING to respond to ligand.

### Effects of WT/SNP heterozygous hSTING on c-di-GMP-dependent and independent IFN-β signaling

**Figure 8:**
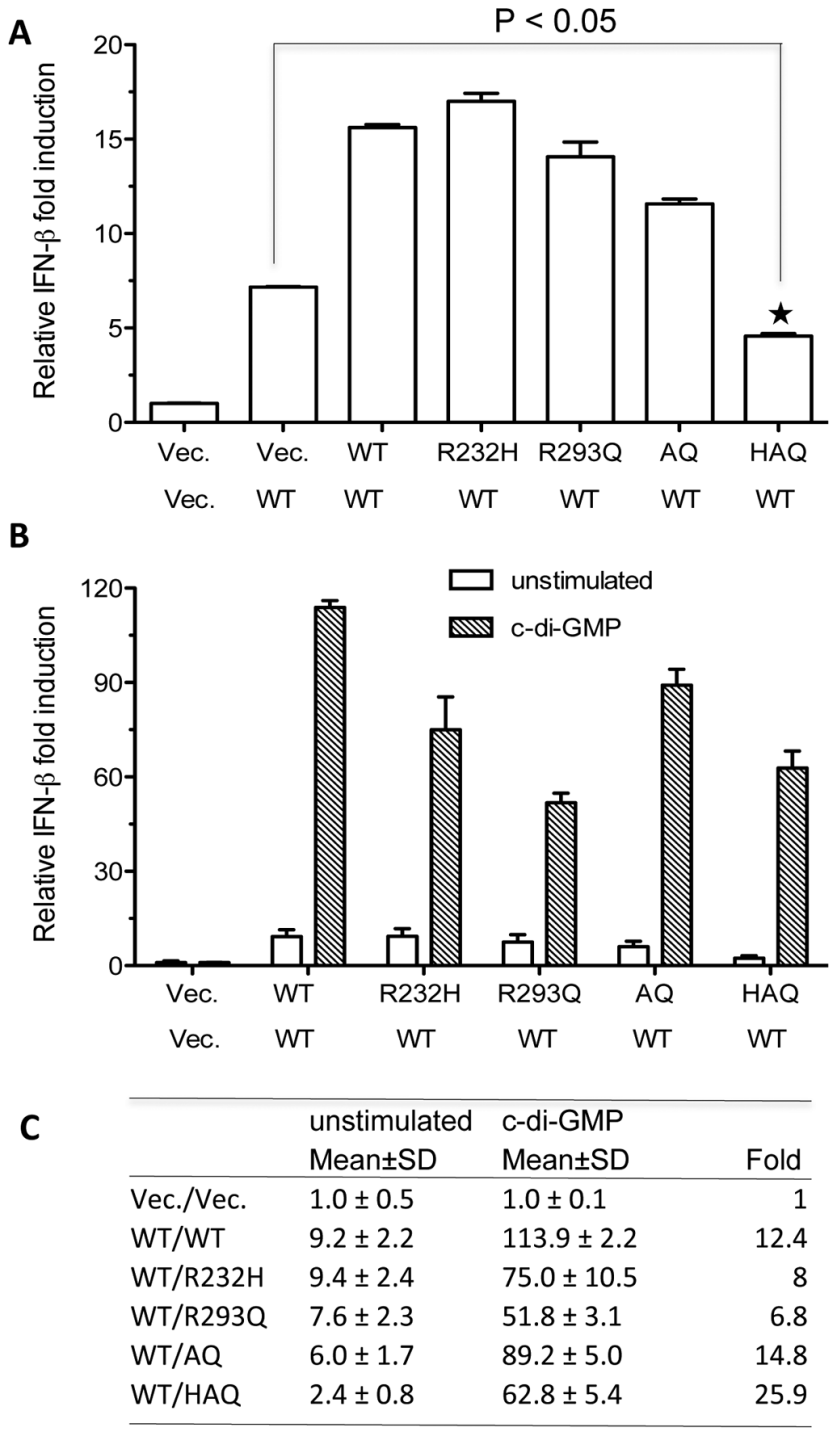
Effects of WT/SNP hSTING heterozygous on c-di-GMP-dependent and independent IFN-β signaling. (A) Effects of transfecting equal amounts of plasmids expressing WT/SNP hSTING on activation of IFN-β reporter. Luciferase activities were determined 30 hours after transfection of the HEK293T cells. (B) Effects of co-transfecting equal amounts of WT/SNP hSTING plasmids on cyclic-di-GMP-mediated activation of IFN-β activity. The cells were first transfected with equal amount of indicated plasmid for 24 h, and then treated by digitonin with or without 20 μg/ml of c-di-GMP. (C) Quantification of relative IFN-β induction in the absence and presence of c-di-GMP ligand. SD denotes the standard derivation. The fold of the further induction by c-di-GMP treatment was shown in the last right column.

We want to determine whether mimicking heterozygosity of WT/SNP could affect the ligand-dependent and independent signaling. HEK293T cells were transiently transfected with plasmid expressing WT hSTING together with equal amount of either empty vector, WT or one of the SNPs (Figure 8A). WT hSTING co-expressed with the R232H or R293Q further enhanced the stimulation of IFN-β promoter activity. However, WT/HAQ heterozygosity significantly decreased the WT hSTING activity (Figure 8A; P<0.005), suggesting that HAQ variant could exhibit a dominant negative effect in the absence of ligand. Next, we tested the heterozygous SNPs in the presence of c-di-GMP. Most of the WT/SNP hSTING decreased the c-di-GMP-dependent activity by 20-50% (Figure 8B and 8C). In contrast, cells co-expressing the WT hSTING and HAQ responded to c-di-GMP by 50% of WT/WT hSTING level. The difference exhibited by the WT/HAQ co-expression in the absence and presence of ligand suggest that a WT/SNP heterozygosity could have lower intrinsic activity, but maintains partial ability to activate innate immune signaling in the presence of ligand. 

### Mechanism for the G230A substitution on signaling

G230 is located in the flexible loop that forms a lid above the c-di-GMP binding pocket in hSTING (Figure 1C). The ability of the G230A STING to respond to lower concentrations of c-di-GMP led us to hypothesize that the G230A mutation could increase the affinity of c-di-GMP binding. 

**Figure 9:**
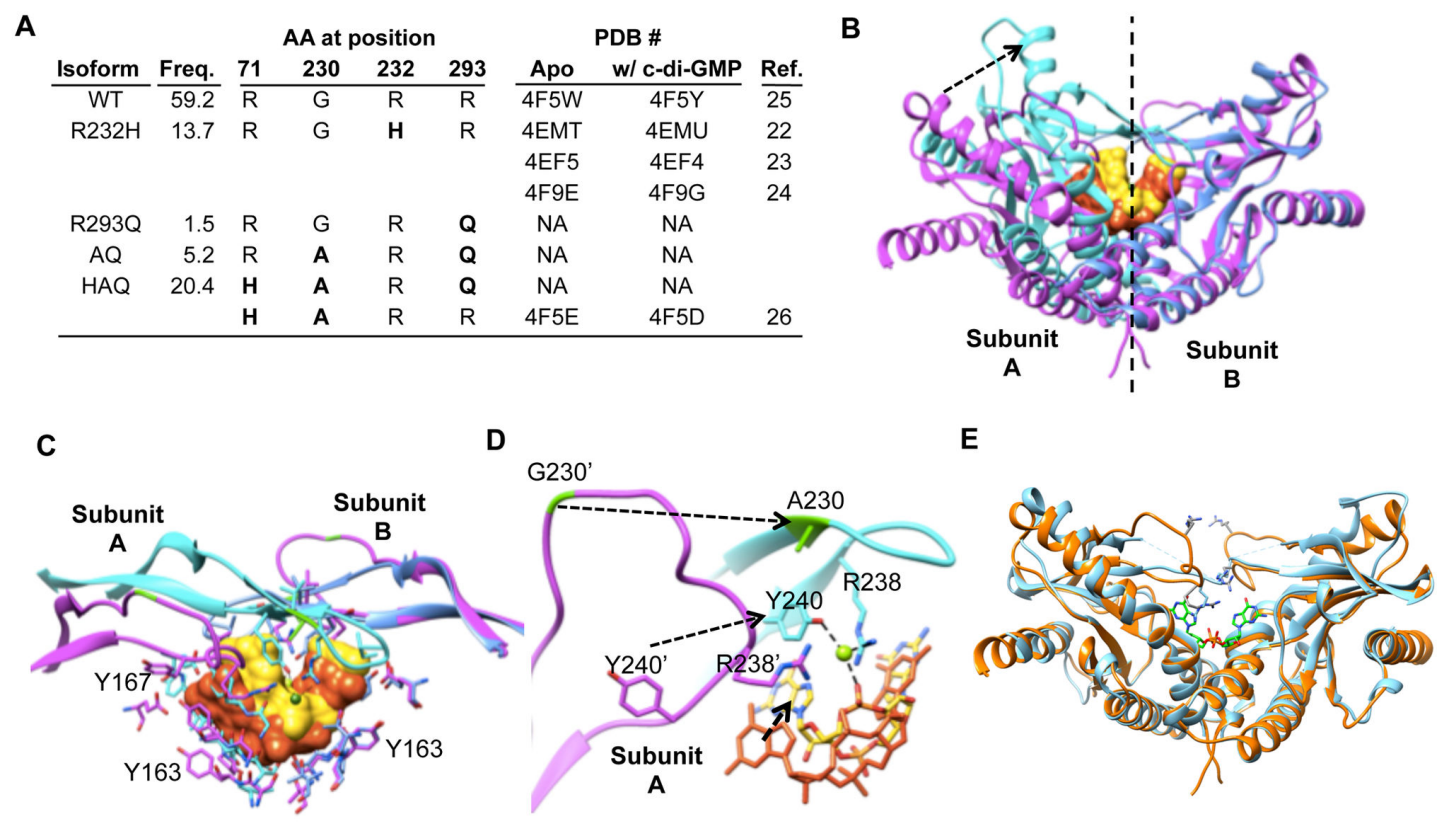
Comparison of the structure of WT and G230A hSTING CTDs complexed to c-di-GMP. (A) A summary of known structures of apo-hSTING CTD and hSTING CTD-c-di-GMP complex. The amino acids substitutions in the structures are labeled in bold. (B) Overlay of G230A-STING:c-di-GMP dimer and the WT-STING:c-di-GMP dimer. The structures are from 4F5E and 4F5D of the PDB. The WT dimer is rendered with magenta ribbon and the c-di-GMP is shown as orange solid surface. The G230A mutant dimer is shown as blue ribbon (cyan: subunit A and blue: subunit B). Compare to all other structures (Figure 1), the subunit A of the ligand bound G230A structure shows the dramatic positional change indicated by an arrow. (C) Closer view of c-di-GMP and the lid region. The loop to ribbon transition in the lid region of the mutant structure is depicted and, within the dimer, Subunit A is located relatively closer to subunit B than in the WT CTD. (D) The conformational change of the lid region of G230A CTD that leads to a more ordered ligand binding. The ligand is shown in orange or yellow sticks and Mg^2+^ is shown as green ball. Residue 230 is colored green. (E) Superposition of Apo WT hSTING (golden brown) and R232H hSTING complexed with c-di-GMPs (cyan).

 To better understand the effect of G230A substitution on hSTING signaling, we analyzed the structures of previously published dimers of the hSTING C-terminal domains (CTDs). A search of the Protein Data Bank yielded the structures of ten CTDs, 5 of which were without ligand (PDB IDs 4EMT, 4F5W, 4EF5, 4F9E and 4F5E) and 5 in complex with c-di-GMP (4EMU, 4F5Y, 4EF4, 4F9E and 4F5D; Figure 9A). Huang et al. [27] have solved the structures of apo-STING and the c-di-GMP-bound CTD with the G230A and the R71H variants but did not discuss the effects of the SNPs (PDB ID 4F5E and 4F5D). Residue 71 was not resolved in the structure and does not lie within the ligand binding CTD.

The superposition of all ten structures revealed that the ligand-bound G230A CTD was the only one that deviated from the others (Figure 9A and Figure S2). All of the other structure, including those without ligand, had roughly comparable global conformations. In contrast, subunit A of ligand-bound G230A CTD structure exhibited a 25° rotation of a helix that connects to the lid motif with respect to subunit B (Figure 9B). This positional change formed a “closed dimer” which brings the lid region of both subunits close to each other [27]. Furthermore, the lid of the ligand bound G230A CTD structure formed two new β strands in each subunit of the dimer (Figure 9C). 

The closed dimer significantly affected the interactions between the ligand and the CTD in the G230A structure. Residues of subunit A are in different positions within the dimer relative to the other structures. In fact, the bound c-di-GMP was tilted towards the β strands in the lid of subunit B to accommodate these positional changes of subunit A within the CTD dimer (Figure 9C and 9D). The alanine side chain of residue 230 within the β-strand formed a hydrophobic cluster under the lid. In addition, residues Y240 and R238 near G230A isoform adopted a positional switch to interact with c-di-GMP that was not observed within the WT CTD (Figure 9D). 

 The structures of the apo-protein and c-di-GMP bound R232H CTD have been reported (Figure 9E). The R232H apo and c-di-GMP bound proteins had root mean square deviations that differ by only 0.69 Å for the 160 c-alpha atoms and 1.26 Å for all atoms. The c-di-GMP-bound complex exhibits a more ordered lid region while the apo-protein had residues that were not resolved. However, the β-strands observed with the lid of the R232H CTD were not formed. These results further confirm that G230A CTD will interact with the ligand in a different way than does WT or the R232H CTD. 

### Biochemical characterization of G230A substitution in the presence of c-di-GMP ligand

**Figure 10:**
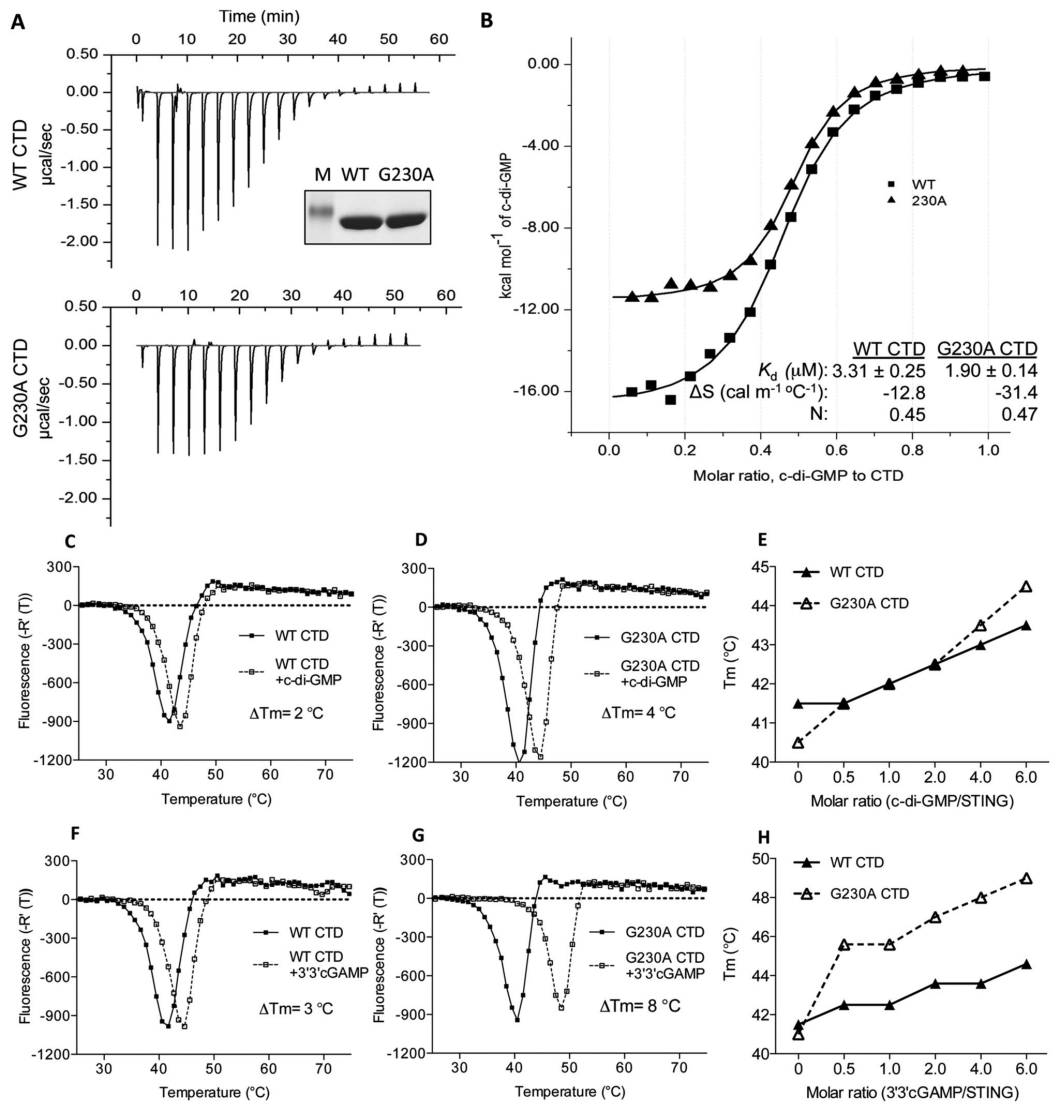
Biochemical analysis of hSTING interaction with c-di-GMP. (A) Isothermal titration calorimetry measurement of the interaction between WT hSTING and c-di-GMP. The recombinant WT hSTING and G230A protein (C-terminal domain, CTD) were purified and quantified by a Nanodrop spectrometer. The purity of proteins was analyzed on SDS-PAGE. ITC was performed at 25 °C as described in the experimental procedures. The dissociation constants and thermodynamic parameters were measured by ITC using a MicroCal iTC200 calorimeter. (B) Overlapping ITC assay of WT and G230A mutant with c-di-GMP. Equilibrium association constant, binding stoichiometry N, enthalpy ΔH were determined by fitting the data based on a single set of identical sites model. (C) Differential scanning fluorimetry assay of WT hSTING with or without c-di-GMP. Where present, c-di-GMP was at 5 molar excess of the apo-protein. The curves represent at least two independent experiments. The ΔT_Mapp_ of hSTING with and without c-di-GMP was shown. The bold line denotes the WT hSTING with H_2_O and the dash line is WT with c-di-GMP. (D) Thermal denaturation of G230A in the absence or presence of 5 molar excess of c-di-GMP. (E) Effects of different molar ratios of c-di-GMP on the T_Mapp_ of the WT or G230A hSTING in differential scanning fluorescence assay. The T_Mapp_ was plotted in the presence of different molar ratios of c-di-GMP. The bold line represents CTD of WT hSTING and dash line is G230A CTD. (F) Differential scanning fluorimetry assay of WT hSTING with or without 3’3’cGAMP. (G) Thermal denaturation of G230A in the absence or presence of 5 molar excess of 3’3’cGAMP. (H) Effects of different molar ratios of 3’3’cGAMP on the T_Mapp_ of the WT or G230A hSTING.

Next, we sought evidence to support the conformational change of the G230A CTD by analyzing the properties of the recombinant protein in solution. We measured the binding affinity of recombinant CTD of WT and G230A hSTING proteins (residues 149-341) by isotheral titration calorimetry. Recombinant G230A CTD bound to c-di-GMP with a dissociation constant (K_d_) 1.9 μM, only slightly better than that of the WT hSTING (K_d_ of 3.3 μM; Figure 10A). However, the thermodynamic parameters of the G230A CTD did exhibit significantly more negative entropic contribution (-31.4 cal/mol) than did the WT CTD (-12.8 cal/mol; Figure 10B). This result indicates that the conformation of G230A hSTING upon c-di-GMP binding was likely to be different than that of the WT hSTING.

To determine whether c-di-GMP binding could induce a more significant conformational change in the G230A CTD, we used differential scanning fluorimetry to monitor the denaturation profile of proteins in the absence or presence of ligand [36]. The denaturation profiles of both the WT and G230A hSTING exhibited a sharp transition in the absence of c-di-GMP and the transition was affected by the presence of c-di-GMP (Figure 10C and 10D). In the absence of c-di-GMP, the midpoint of the transition, T_Mapp,_ was 41.5°C for the WT CTD, 1°C higher than the G230A. In the presence of 5 molar excess of c-di-GMP, the T_Mapp_ of the WT STING increased by 2°C while the T_Mapp_ of the G230A hSTING was increased by 4°C (Figure 10C and 10D). The changes in the WT and G230A hSTING were also observed over a range of c-di-GMP concentrations (Figure 10E). Comparable results were observed with 3’3’ cGAMP ligand. The presence of 5 molar excess of 3’3’ cGAMP increased the T_Mapp_ of WT hSTING by 3°C while the T_Mapp_ of the G230A hSTING was increased by 8°C (Figure 10F-H). These results are consistent with functional results that the G230A hSTING can activate signal transduction upon c-di-GMP binding and the conformational change exhibited by the structure of the G230A hSTING. 

## Discussion

Single nucleotide polymorphisms in innate immune receptors could affect the production of type I interferon and cytokines [30,37] that could lead to altered frequencies of human diseases [32,38-40]. In this work, we analyzed the SNPs of hSTING derived from 1000 Genome Project and examined their abilities to activate IFN-β and NF-κB reporter in the reporter assay. We found that all except one of the changes (R232H) decreased the ability of the SNPs to activate IFN-β or the NF-κb promoters in the absence of exogenous ligands. Furthermore, in the presence of the cyclic dinucleotides, all of the variants had either modest or severe decrease in the activation of the IFN-β or NF-κb promoters (Table 2). Given that several of the SNPs in hSTING are present in 1 to ~20% of the human population, these results raises the possibility that there may be selection for reduced signaling by the hSTING protein. In addition, we found that the HAQ haplotype that co-segregated in ~20% of the human population in the 1000 Genome Project consists of two substitutions, R71H and R293Q that decreased basal and ligand-induced STING signaling, but that substitution G230A enhanced hSTING signaling in response to ligands. The G230A substitution lies in the lid portion of the c-di-GMP binding pocket and renders STING into a more sensitive responder to lower concentration of ligand, likely by causing altered binding of c-di-GMP. 

Our results are consistent with previous observations by Jin et al. [28] who identified that HAQ is a loss-of-function haplotype of hSTING and that HAQ had a dominant negative effect when co-expressed with the WT hSTING. Although both R293Q and G230A substitutions might contribute to the decreased stimulation, R71 is primarily responsible for this reduction since an R71H substitution decreased the IFN-β stimulation to 10-20% of the WT level. The hSTING is ER-targeted protein with four putative transmembrane domains in the N-terminus [6]. R71 is located between the transmembrane helix 2 and helix 3. The substitution of R71H could affect its localization and result in the defect in innate immune signaling. 

In the presence of c-di-GMP, R232H substitution decreased the further response to ligand by 30-50%. This result differs from previous work on the mouse STING [13], where R231 of mSTING (corresponding to R232 of hSTING), was critical for the response to c-di-GMP. Mutation of R231 to alanine could result in the loss of the ability of mSTING to stimulate IFN-β production in 293T cells [13], suggesting that the recognition of c-di-GMP ligand between human and mouse STING proteins could be distinct. Indeed, differential recognition of anticancer reagents 5,6-dimethylxanthenone-4-acetic acid (DMXAA) and flavonoids by hSTING and mSTING was recently reported [41-43]. The DMXAA only bound to mSTING but not hSTING to induce type I IFNs in mice [41,42]. Furthermore, the small-molecular antiviral compound, 10-carboxymethyl-9-acridanone mediated IFN production in mice system but not in human cells due to its inability to bind to hSTING [44]. Interestingly, we also observed that hSTING R232H was defective for sensing c-di-AMP and 3’3’ cGAMP (Figure 4B, 4C), indicating that hSTING might also differentially recognize the c-di-GMP and c-di-AMP. It will be of interest to further examine how the R232H substitution could differentially affect the specificity of cyclic dinucleotide recognition by hSTING. 

The R293Q substitution was present in about 30% of the human population when the frequencies from R293Q, AQ and HAQ are added (Figure 1). Although a R293Q homozygous only modest decreased the ligand-independent signaling from the IFN-β and NF-κB reporters in reporter assays, it was severely defective for the response to c-di-GMP ligand. Unlike residue R232, R293 does not directly contact with c-di-GMP ligand (Figure 1C) in either the opened or closed conformation of STING/c-di-GMP complex [23,27]. Currently, we don’t know how the R293 and the consequence of the R293Q substitution could affect signal transduction by hSTING. Possible effects are on the oligomerization of hSTING and/or the recruitment and activation of TBK1 kinase [11].

The HAQ variant can respond to c-di-GMP ligand at 40-80% of the level of WT hSTING, depended on the concentration of protein expressed. At lower hSTING protein levels, HAQ has about 80% of WT level, however, higher concentration of HAQ protein did not result in commensurate higher levels of signaling (Figure 6C). Jin et al. [28] found that bacterium *Listeria monocytogenes* infection resulted in IFN-β induction being impaired to about 10-20% of WT level in cells stable expressing HAQ haplotype. This difference could be due to the higher hSTING expression in the stable cell line. Furthermore, since STING can sense bacterial DNA or RNA to trigger type I interferon response, it is possible that HAQ haplotype could also affect DNA or RNA-mediated signal pathway in ways distinct from c-di-GMP. 

The ability of HAQ isoform to respond to c-di-GMP ligand is primarily due to the G230A substitution, because it significantly enhances the response to cyclic dinucleotides. The recombinant G230A hSTING CTD exhibits only slightly better binding affinity with c-di-GMP than does WT hSTING in the isothermal calorimetry experiment, suggesting that G230A has only a modest effect on the binding affinity with c-di-GMP. However, It is possible that the population of hSTING exists in equilibrium between the open and closed dimers and that the G230A substitutions favored the closed conformation in the presence of ligand. It is also possible that other domains of hSTING could contribute to improved ligand recognition. Nonetheless, the G230A hSTING CTD exists in a more flexible state relative to the WT and this increased flexibility facilitates the changes observed when the G230A CTD was complexed to c-di-GMP. 

The most parsimonious lineage for the G230A substitution showed that it arose after the R293Q variation during the derivation of the HAQ haplotype, suggesting that this change could link to improve ligand-dependent signaling. One interpretation of the high abundance of this HAQ haplotype may be that it is advantageous to have decreased hSTING signaling in the absence of ligand, but to also have more ligand-responsive hSTING in the presence of cyclic dinucleotide ligands. 

## Supporting Information

Figure S1 **Effects of substitutions in the HAQ variant on c-di-GMP- or 3’3’cGAMP-dependent NF-kB signaling.** (A) c-di-GMP-mediated NF-kB signaling. 2 ng of each plasmid was transfected into HEK293T cells for 24 h and then the cells were mock-stimulated or stimulated with 20 μg/ml of c-di-GMP. (B) Effects of substitutions on cGAMP-mediated NF-kB signaling. 2 ng of each plasmid was transfected into HEK293T cells for 24 h prior to mock-transfection or super-transfection with 20 μg/ml of cGAMP. (C) G230A can promote phosphorylation of IRF3 at levels higher than WT STING. The Loading control is a nonspecific band that is recognized by the anti-body to STING.
(TIFF)

Figure S2 **Structural comparison of hSTING.** (A) A comparison of 10 different crystal structures of STING available in the PDB (both apo and c-di-GMP bound forms are included). The arrow mark in each subunit indicates the location of the G230 residue, in context to the ligand and dimer interface. The comparison also depicts the flexibility of the loops associated with G230 residue, within the dimer. (B) Superimposition of apo-G230A STING structure and apo-STING (G230). Comparison of the crystal structure of apo-hSTING (G230; orange – PDB ID 4f5w) with that of apo-G230A STING (Blue- PDB ID 4f5E). The ribbons are rendered based on average B-factor of individual residues and show distinct flexibility and dynamic nature of the loop associated with the residue 230.
(TIFF)

## References

[1] Takeuchi O, Akira S (2010) Pattern recognition receptors and inflammation. Cell 140: 805-820. doi:10.1016/j.cell.2010.01.022. PubMed: 20303872. 20303872

[2] Zhang Z, Yuan B, Bao M, Lu N, Kim T et al. (2011) The helicase DDX41 senses intracellular DNA mediated by the adaptor STING in dendritic cells. Nat Immunol 12: 959-965. doi:10.1038/ni.2091. PubMed: 21892174. 21892174 PMC3671854

[3] Unterholzner L, Keating SE, Baran M, Horan KA, Jensen SB et al. (2010) IFI16 is an innate immune sensor for intracellular DNA. Nat Immunol 11: 997-1004. doi:10.1038/ni.1932. PubMed: 20890285. 20890285 PMC3142795

[4] Fernandes-Alnemri T, Yu JW, Datta P, Wu J, Alnemri ES (2009) AIM2 activates the inflammasome and cell death in response to cytoplasmic DNA. Nature 458: 509-513. doi:10.1038/nature07710. PubMed: 19158676. 19158676 PMC2862225

[5] Takaoka A, Wang Z, Choi MK, Yanai H, Negishi H et al. (2007) DAI (DLM-1/ZBP1) is a cytosolic DNA sensor and an activator of innate immune response. Nature 448: 501-505. doi:10.1038/nature06013. PubMed: 17618271. 17618271

[6] Ishikawa H, Barber GN (2008) STING is an endoplasmic reticulum adaptor that facilitates innate immune signalling. Nature 455: 674-678. doi:10.1038/nature07317. PubMed: 18724357. 18724357 PMC2804933

[7] Zhong B, Yang Y, Li S, Wang YY, Li Y et al. (2008) The adaptor protein MITA links virus-sensing receptors to IRF3 transcription factor activation. Immunity 29: 538-550. doi:10.1016/j.immuni.2008.09.003. PubMed: 18818105. 18818105

[8] Sun W, Li Y, Chen L, Chen H, You F et al. (2009) ERIS, an endoplasmic reticulum IFN stimulator, activates innate immune signaling through dimerization. Proc Natl Acad Sci U S A 106: 8653-8658. doi:10.1073/pnas.0900850106. PubMed: 19433799. 19433799 PMC2689030

[9] Burdette DL, Vance RE (2013) STING and the innate immune response to nucleic acids in the cytosol. Nat Immunol 14: 19-26. PubMed: 23238760. 23238760 10.1038/ni.2491

[10] Ishikawa H, Ma Z, Barber GN (2009) STING regulates intracellular DNA-mediated, type I interferon-dependent innate immunity. Nature 461: 788-792. doi:10.1038/nature08476. PubMed: 19776740. 19776740 PMC4664154

[11] Tanaka Y, Chen ZJ (2012) STING specifies IRF3 phosphorylation by TBK1 in the cytosolic DNA signaling pathway. Sci Signal 5: ra20–: ra20 PubMed: 22394562. 10.1126/scisignal.2002521 PMC3549669 22394562

[12] Jin L, Hill KK, Filak H, Mogan J, Knowles H et al. (2011) MPYS is required for IFN response factor 3 activation and type I IFN production in the response of cultured phagocytes to bacterial second messengers cyclic-di-AMP and cyclic-di-GMP. J Immunol 187: 2595-2601. doi:10.4049/jimmunol.1100088. PubMed: 21813776. 21813776 PMC3159690

[13] Burdette DL, Monroe KM, Sotelo-Troha K, Iwig JS, Eckert B et al. (2011) STING is a direct innate immune sensor of cyclic di-GMP. Nature 478: 515-518. doi:10.1038/nature10429. PubMed: 21947006. 21947006 PMC3203314

[14] Parvatiyar K, Zhang Z, Teles RM, Ouyang S, Jiang Y et al. (2012) The helicase DDX41 recognizes the bacterial secondary messengers cyclic di-GMP and cyclic di-AMP to activate a type I interferon immune response. Nat Immunol 13: 1155-1161. doi:10.1038/ni.2460. PubMed: 23142775. 23142775 PMC3501571

[15] Wu J, Sun L, Chen X, Du F, Shi H et al. (2013) Cyclic GMP-AMP is an endogenous second messenger in innate immune signaling by cytosolic DNA. Science 339: 826-830. doi:10.1126/science.1229963. PubMed: 23258412. 23258412 PMC3855410

[16] Sun L, Wu J, Du F, Chen X, Chen ZJ (2013) Cyclic GMP-AMP synthase is a cytosolic DNA sensor that activates the type I interferon pathway. Science 339: 786-791. doi:10.1126/science.1232458. PubMed: 23258413. 23258413 PMC3863629

[17] Davies BW, Bogard RW, Young TS, Mekalanos JJ (2012) Coordinated regulation of accessory genetic elements produces cyclic di-nucleotides for V. cholerae virulence. Cell 149: 358-370. doi:10.1016/j.cell.2012.01.053. PubMed: 22500802. 22500802 PMC3620040

[18] Gao P, Ascano M, Wu Y, Barchet W, Gaffney BL et al. (2013) Cyclic [G(2',5')pA(3',5')p] is the metazoan second messenger produced by DNA-activated cyclic GMP-AMP synthase. Cell 153: 1094-1107. doi:10.1016/j.cell.2013.04.046. PubMed: 23647843. 23647843 PMC4382009

[19] Ablasser A, Goldeck M, Cavlar T, Deimling T, Witte G et al. (2013) cGAS produces a 2'-5'-linked cyclic dinucleotide second messenger that activates STING. Nature 498: 380-384. doi:10.1038/nature12306. PubMed: 23722158. 23722158 PMC4143541

[20] Diner EJ, Burdette DL, Wilson SC, Monroe KM, Kellenberger CA et al. (2013) The innate immune DNA sensor cGAS produces a noncanonical cyclic dinucleotide that activates human STING. Cell Rep 3: 1355-1361. doi:10.1016/j.celrep.2013.05.009. PubMed: 23707065. 23707065 PMC3706192

[21] Zhang X, Shi H, Wu J, Zhang X, Sun L et al. (2013) Cyclic GMP-AMP Containing Mixed Phosphodiester Linkages Is An Endogenous High-Affinity Ligand for STING. Mol Cell 51: 226-235. doi:10.1016/j.molcel.2013.05.022. PubMed: 23747010. 23747010 PMC3808999

[22] Gall A, Treuting P, Elkon KB, Loo YM, Gale M Jr. et al. (2012) Autoimmunity initiates in nonhematopoietic cells and progresses via lymphocytes in an interferon-dependent autoimmune disease. Immunity 36: 120-131. doi:10.1016/j.immuni.2011.11.018. PubMed: 22284419. 22284419 PMC3269499

[23] Shu C, Yi G, Watts T, Kao CC, Li P (2012) Structure of STING bound to cyclic di-GMP reveals the mechanism of cyclic dinucleotide recognition by the immune system. Nat Struct Mol Biol 19: 722-724. doi:10.1038/nsmb.2331. PubMed: 22728658. 22728658 PMC3392545

[24] Ouyang S, Song X, Wang Y, Ru H, Shaw N et al. (2012) Structural analysis of the STING adaptor protein reveals a hydrophobic dimer interface and mode of cyclic di-GMP binding. Immunity 36: 1073-1086. doi:10.1016/j.immuni.2012.03.019. PubMed: 22579474. 22579474 PMC3654694

[25] Yin Q, Tian Y, Kabaleeswaran V, Jiang X, Tu D et al. (2012) Cyclic di-GMP sensing via the innate immune signaling protein STING. Mol Cell 46: 735-745. doi:10.1016/j.molcel.2012.05.029. PubMed: 22705373. 22705373 PMC3697849

[26] Shang G, Zhu D, Li N, Zhang J, Zhu C et al. (2012) Crystal structures of STING protein reveal basis for recognition of cyclic di-GMP. Nat Struct Mol Biol 19: 725-727. doi:10.1038/nsmb.2332. PubMed: 22728660. 22728660

[27] Huang YH, Liu XY, Du XX, Jiang ZF, Su XD (2012) The structural basis for the sensing and binding of cyclic di-GMP by STING. Nat Struct Mol Biol 19: 728-730. doi:10.1038/nsmb.2333. PubMed: 22728659. 22728659

[28] Jin L, Xu LG, Yang IV, Davidson EJ, Schwartz DA et al. (2011) Identification and characterization of a loss-of-function human MPYS variant. Genes Immun 12: 263-269. doi:10.1038/gene.2010.75. PubMed: 21248775. 21248775 PMC3107388

[29] 1000 Genomes Project Consortium, Abecasis GR, Altshuler D, Auton A, Brooks LD, et al. (2010) A map of human genome variation from population-scale sequencing. Nature 467: 1061-1073. doi:10.1038/nature09534. PubMed: 20981092. 20981092 PMC3042601

[30] Ranjith-Kumar CT, Miller W, Sun J, Xiong J, Santos J et al. (2007) Effects of single nucleotide polymorphisms on Toll-like receptor 3 activity and expression in cultured cells. J Biol Chem 282: 17696-17705. doi:10.1074/jbc.M700209200. PubMed: 17434873. 17434873

[31] Woodward JJ, Iavarone AT, Portnoy DA (2010) c-di-AMP secreted by intracellular Listeria monocytogenes activates a host type I interferon response. Science 328: 1703-1705. doi:10.1126/science.1189801. PubMed: 20508090. 20508090 PMC3156580

[32] Netea MG, Wijmenga C, O'Neill LA (2012) Genetic variation in Toll-like receptors and disease susceptibility. Nat Immunol 13: 535-542. doi:10.1038/ni.2284. PubMed: 22610250. 22610250

[33] Pothlichet J, Quintana-Murci L (2013) The genetics of innate immunity sensors and human disease. Int Rev Immunol 32: 157-208. doi:10.3109/08830185.2013.777064. PubMed: 23570315. 23570315

[34] Sauer JD, Sotelo-Troha K, von Moltke J, Monroe KM, Rae CS et al. (2011) The N-ethyl-N-nitrosourea-induced Goldenticket mouse mutant reveals an essential function of Sting in the in vivo interferon response to Listeria monocytogenes and cyclic dinucleotides. Infect Immun 79: 688-694. doi:10.1128/IAI.00999-10. PubMed: 21098106. 21098106 PMC3028833

[35] Oeckinghaus A, Hayden MS, Ghosh S (2011) Crosstalk in NF-kB signaling pathways. Nat Immunol: 696-708. 10.1038/ni.2065 21772278

[36] Niesen FH, Berglund H, Vedadi M (2007) The use of differential scanning fluorimetry to detect ligand interactions that promote protein stability. Nat Protoc 2: 2212-2221. doi:10.1038/nprot.2007.321. PubMed: 17853878. 17853878

[37] Shigemoto T, Kageyama M, Hirai R, Zheng J, Yoneyama M et al. (2009) Identification of loss of function mutations in human genes encoding RIG-I and MDA5: implications for resistance to type I diabetes. J Biol Chem 284: 13348-13354. doi:10.1074/jbc.M809449200. PubMed: 19324880. 19324880 PMC2679434

[38] Smyth DJ, Cooper JD, Bailey R, Field S, Burren O et al. (2006) A genome-wide association study of nonsynonymous SNPs identifies a type 1 diabetes locus in the interferon-induced helicase (IFIH1) region. Nat Genet 38: 617-619. doi:10.1038/ng1800. PubMed: 16699517. 16699517

[39] Nejentsev S, Walker N, Riches D, Egholm M, Todd JA (2009) Rare variants of IFIH1, a gene implicated in antiviral responses, protect against type 1 diabetes. Science 324: 387-389. doi:10.1126/science.1167728. PubMed: 19264985. 19264985 PMC2707798

[40] Zhang SY, Jouanguy E, Ugolini S, Smahi A, Elain G et al. (2007) TLR3 deficiency in patients with herpes simplex encephalitis. Science 317: 1522-1527. doi:10.1126/science.1139522. PubMed: 17872438. 17872438

[41] Conlon J, Burdette DL, Sharma S, Bhat N, Thompson M et al. (2013) Mouse, but not human STING, binds and signals in response to the vascular disrupting agent 5,6-dimethylxanthenone-4-acetic acid. J Immunol 190: 5216-5225. doi:10.4049/jimmunol.1300097. PubMed: 23585680. 23585680 PMC3647383

[42] Prantner D, Perkins DJ, Lai W, Williams MS, Sharma S et al. (2012) 5,6-Dimethylxanthenone-4-acetic acid (DMXAA) activates stimulator of interferon gene (STING)-dependent innate immune pathways and is regulated by mitochondrial membrane potential. J Biol Chem 287: 39776-39788. doi:10.1074/jbc.M112.382986. PubMed: 23027866. 23027866 PMC3501038

[43] Kim S, Li L, Maliga Z, Yin Q, Wu H et al. (2013) Anticancer Flavonoids Are Mouse-Selective STING Agonists. ACS Chem Biol. PubMed: 23683494 10.1021/cb400264n PMC3815523 23683494

[44] Cavlar T, Deimling T, Ablasser A, Hopfner KP, Hornung V (2013) Species-specific detection of the antiviral small-molecule compound CMA by STING. EMBO J 32: 1440-1450. doi:10.1038/emboj.2013.86. PubMed: 23604073. 23604073 PMC3655471

[45] Tamura K, Peterson D, Peterson N, Stecher G, Nei M et al. (2011) MEGA5: molecular evolutionary genetics analysis using maximum likelihood, evolutionary distance, and maximum parsimony methods. Mol Biol Evol 28: 2731-2739. doi:10.1093/molbev/msr121. PubMed: 21546353. 21546353 PMC3203626

