# Supplementary figures and images for: Single Nucleotide Polymorphisms of Human STING Can Affect Innate Immune Response to Cyclic Dinucleotides
Source: PLoS One. 2013 Oct 21;8(10):e77846. doi: 10.1371/journal.pone.0077846 (PMC3804601; doi:10.1371/journal.pone.0077846)

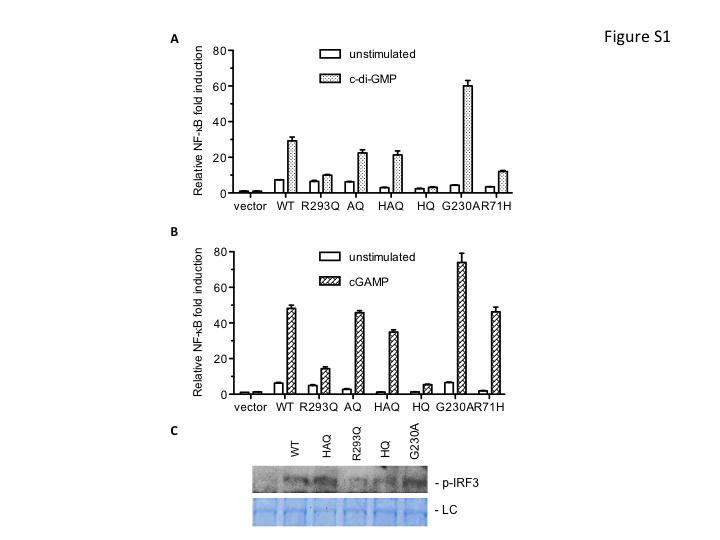

Supplement: Figure S1 — Effects of substitutions in the HAQ variant on c-di-GMP- or 3’3’cGAMP-dependent NF-kB signaling. (A) c-di-GMP-mediated NF-kB signaling. 2 ng of each plasmid was transfected into HEK293T cells for 24 h and then the cells were mock-stimulated or stimulated with 20 μg/ml of c-di-GMP. (B) Effects of substitutions on cGAMP-mediated NF-kB signaling. 2 ng of each plasmid was transfected into HEK293T cells for 24 h prior to mock-transfection or super-transfection with 20 μg/ml of cGAMP. (C) G230A can promote phosphorylation of IRF3 at levels higher than WT STING. The Loading control is a nonspecific band that is recognized by the anti-body to STING. (TIFF) [file pone.0077846.s001.tiff]

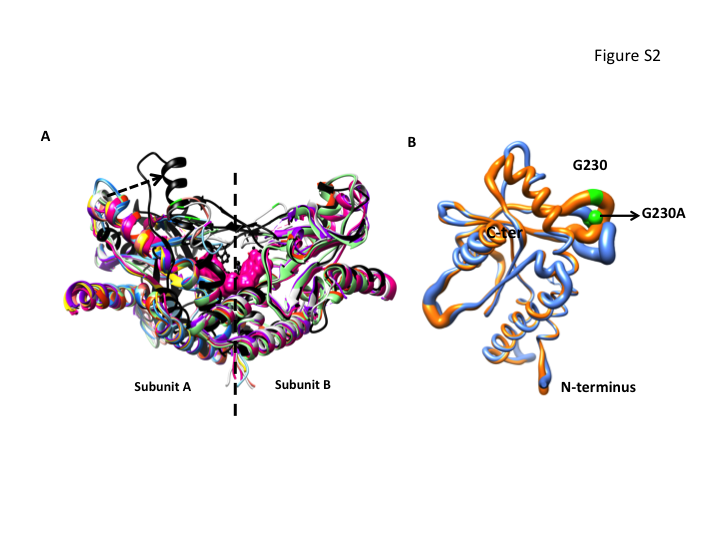

Supplement: Figure S2 — Structural comparison of hSTING. (A) A comparison of 10 different crystal structures of STING available in the PDB (both apo and c-di-GMP bound forms are included). The arrow mark in each subunit indicates the location of the G230 residue, in context to the ligand and dimer interface. The comparison also depicts the flexibility of the loops associated with G230 residue, within the dimer. (B) Superimposition of apo-G230A STING structure and apo-STING (G230). Comparison of the crystal structure of apo-hSTING (G230; orange – PDB ID 4f5w) with that of apo-G230A STING (Blue- PDB ID 4f5E). The ribbons are rendered based on average B-factor of individual residues and show distinct flexibility and dynamic nature of the loop associated with the residue 230. (TIFF) [file pone.0077846.s002.tiff]
